# IntrAnat Electrodes: A Free Database and Visualization Software for Intracranial Electroencephalographic Data Processed for Case and Group Studies

**DOI:** 10.3389/fninf.2018.00040

**Published:** 2018-07-06

**Authors:** Pierre Deman, Manik Bhattacharjee, François Tadel, Anne-Sophie Job, Denis Rivière, Yann Cointepas, Philippe Kahane, Olivier David

**Affiliations:** ^1^Inserm, U1216, Grenoble Institut des Neurosciences (GIN), Grenoble, France; ^2^Grenoble Institut des Neurosciences (GIN), University of Grenoble Alpes, Grenoble, France; ^3^Neurology Department, Grenoble-Alpes University Hospital, Grenoble, France; ^4^Neurospin—CEA Saclay, UNATI Lab, Gif sur Yvette, France; ^5^Multicenter Neuroimaging Platform, CATI, Paris, France

**Keywords:** stereoelectroencephalography, epilepsy surgery, Python software, multimodal neuroimaging, database and tools development

## Abstract

In some cases of pharmaco-resistant and focal epilepsies, intracranial recordings performed epidurally (electrocorticography, ECoG) and/or in depth (stereoelectroencephalography, SEEG) can be required to locate the seizure onset zone and the eloquent cortex before surgical resection. In SEEG, each electrode contact records brain’s electrical activity in a spherical volume of 3 mm diameter approximately. The spatial coverage is around 1% of the brain and differs between patients because the implantation of electrodes is tailored for each case. Group studies thus need a large number of patients to reach a large spatial sampling, which can be achieved more easily using a multicentric approach such as implemented in our F-TRACT project (f-tract.eu). To facilitate group studies, we developed a software—IntrAnat Electrodes—that allows to perform virtual electrode implantation in patients’ neuroanatomy and to overlay results of epileptic and functional mapping, as well as resection masks from the surgery. IntrAnat Electrodes is based on a patient database providing multiple search criteria to highlight various group features. For each patient, the anatomical processing is based on a series of software publicly available. Imaging modalities (Positron Emission Tomography (PET), anatomical MRI pre-implantation, post-implantation and post-resection, functional MRI, diffusion MRI, Computed Tomography (CT) with electrodes) are coregistered. The 3D T1 pre-implantation MRI gray/white matter is segmented and spatially normalized to obtain a series of cortical parcels using different neuroanatomical atlases. On post-implantation images, the user can position 3D models of electrodes defined by their geometry. Each electrode contact is then labeled according to its position in the anatomical atlases, to the class of tissue (gray or white matter, cerebro-spinal fluid) and to its presence inside or outside the resection mask. Users can add more functionally informed labels on contact, such as clinical responses after electrical stimulation, cortico-cortical evoked potentials, gamma band activity during cognitive tasks or epileptogenicity. IntrAnat Electrodes software thus provides a means to visualize multimodal data. The contact labels allow to search for patients in the database according to multiple criteria representing almost all available data, which is to our knowledge unique in current SEEG software. IntrAnat Electrodes will be available in the forthcoming release of BrainVisa software and tutorials can be found on the F-TRACT webpage.

## Introduction

In some cases of pharmaco-resistant and focal epilepsies, intracranial recordings performed epidurally (electrocorticography, ECoG; Fernández and Loddenkemper, 2013) and/or in depth (stereoelectroencephalography, SEEG; Kahane and Dubeau, 2014) can be required to locate the seizure onset zone and the eloquent cortex. The following resective surgery is applicable only if the epileptogenic zone (EZ) is found focal, well delineated, and safely removable.

The SEEG methodology consists in the implantation of around 12 deep intracerebral electrodes during a few days (usually one or 2 weeks), corresponding to approximately 120 electrodes contacts recorded in total. Each contact records the electrical activity in a spherical volume of 3 mm diameter approximately, leading to a spatial coverage of only around 1% of the brain activity per patient (Halgren et al., 1998; Lachaux et al., 2003). Therefore, the implantation strategy is tailored for each patient in order to maximize the chances to record the suspected EZ and its network of propagation. The hypothesis on the EZ is made from the clinical history of the patient, his/her MRI (often T1 and Fluid-Attenuated Inversion Recovery (FLAIR), sometimes Diffusion Tensor Imaging (DTI), fMRI), his interictal Positron Emission Tomography (PET) metabolism and video-EEG monitoring. When the patient is selected as a candidate for SEEG exploration, much medical information that will be useful to interpret the SEEG signals is thus already available. The positioning of electrode contacts on the pre-implantation data is therefore very important (Arnulfo et al., 2015b; Wang et al., 2016; Narizzano et al., 2017; Vakharia et al., 2017). A limited number of electrodes can be implanted, their trajectories being constrained by the blood vessel organization which is patient-specific (Rodionov et al., 2013; Zuluaga et al., 2014) and adapted to the EZ hypothesis from pre-implantation data (Narizzano et al., 2017). The brain is thus sampled differently between patients, and the final surgical resection may differ, even for similar types of epilepsy.

During SEEG exploration, recordings of different processes are obtained, e.g., resting state oscillations, epileptic seizures, responses to cognitive tasks and to cortical stimulations. For group studies on the reproducibility of those signals, a large number of patients is therefore needed, involving multicentric data pooled over long periods. To facilitate these studies, we developed a research software called IntrAnat Electrodes, which is free and open-source under the GNU-GPL license, coded in Python and based on software largely distributed within the neuroimaging community (SPM, ANTs, Freesurfer, BrainVisa). It can be used for research purposes only, and manages almost every patients’ anonymized data (except genetic information and videos), allowing visualization of electrodes and electrode contacts over many imaging modalities (T1, T2, FLAIR, Fast Gray Matter Acquisition Inversion Recovery (F-GATIR), DTI tractography, PET, CT), segmentation of gray and white matter from the 3D T1, and using multiple parcellations of the brain in its native space—MarsAtlas (Auzias et al., 2013; Avants et al., 2011a), Destrieux (Destrieux et al., 2010)—or in the MNI referential—Brodmann (Brodmann, 1909), MaxProbMap (Hammers et al., 2003)—to label electrode contacts. IntrAnat Electrodes allows to search across patients according to a variety of anatomical criteria, such as the location of recordings or the presence of a resection in a particular region, and of functional criteria, such as whether electrical stimulation of a contact in a given parcel led to a clinical response or whether a modification of the gamma-band amplitude was elicited by a cognitive task.

An important part of the current project has been to make the interface user-friendly, allowing its use for research studies by neurologists and neurosurgeons without specialized training. IntrAnat Electrodes is based on BrainVisa^1^ and developed as a BrainVisa toolbox. This allows the use of all functionality proposed by the community, mainly MarsAtlas parcellation (Auzias et al., 2016), Morphologist for segmentation and sulci recognition^2^ and Freesurfer for alternate segmentation (Dale et al., 1999; Fischl et al., 1999). Existing software for electrodes positioning—Jimbo Dicom Viewer^3^, SEEG Assistant (Narizzano et al., 2017), EpiNav (Rodionov et al., 2013; Vakharia et al., 2017), BFM (Wang et al., 2016), iELVis (Groppe et al., 2017)—allow mainly to precisely position the electrodes, using the coregistered 3D pre and post-implantation images and other coregistered 3D imaging modalities. As a BrainVisa toolbox, IntrAnat Electrodes uses data organized in a SQLite^4^ database making group studies easier, and allows combining and displaying a large amount of data for each subject leading to better insights from the available data.

The software source code is available here: https://github.com/IntrAnatSEEGSoftware/IntrAnat with a basic documentation here: https://github.com/IntrAnatSEEGSoftware/IntrAnat/wiki. A docker image for Intranat Electrodes including all required dependencies is available on the public docker repository: https://store.docker.com/community/images/demanp/intranat. Please use the “release” tag to download the stable version (docker pull demanp/intranat:release).

## Methods

The software is distributed under GNU GPL license, it is free and open-source. It can be used for research purposes only. More documentation and tutorials can be found at: https://f-tract.eu/software/intranat/. The data used here to illustrate the software characteristics were acquired for strict clinical reasons. They were retrospectively collected for research as part of the protocol INSERM IRB 14-140 which was approved by the International Review Board at INSERM, Paris, France. All patients gave written informed consent in accordance with the Declaration of Helsinki.

### Summary

**Figure 1:**
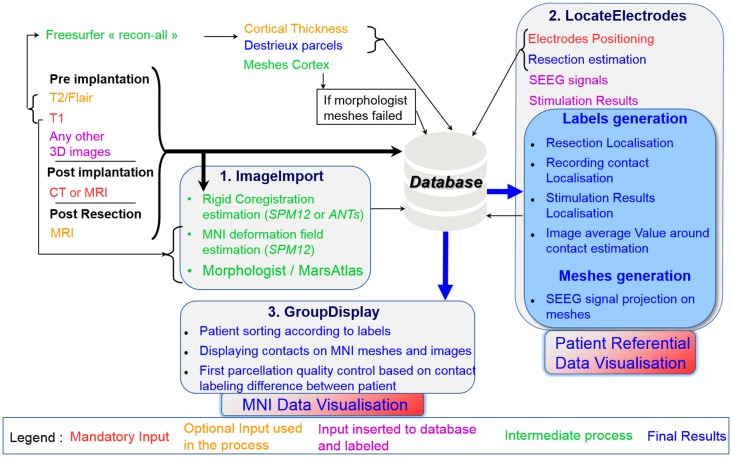
Summary of the data importation and workflow of IntrAnat Electrodes with the three graphical user interface (GUI) interfaces.

IntrAnat software is composed of three different interfaces: ImageImport, LocateElectrodes and GroupDisplay, that are described in respective parts of the methods. Figure 1 summarizes the software and data organization.

Briefly, ImageImport allows the user to import all 3D images and run the coregistration between them, compute the deformation field to the MNI, and performs segmentation/parcellation of the 3D image pre-implantation using MarsAtlas or/and Freesurfer. LocateElectrodes allows to position electrodes and to label their contacts according to multiple criteria (anatomical or SEEG analysis results, such as presence of a response during a specific cognitive task). GroupDisplay allows to select patients according to criteria from contact and resection labeling done in LocateElectrodes and can display data from those patients in a common template.

### First Interface: ImageImport

**Figure 2:**
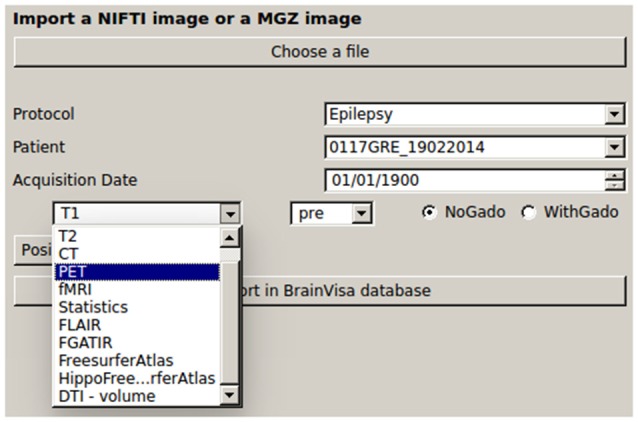
GUI for importing the data in IntrAnat Electrodes. BrainVisa database is organized in terms of Protocol, Patient code and acquisition date. The left panel shows the possible classes of imported images, which can be specified as “pre,” “post,” and “postop” according to the (stereoelectroencephalography, SEEG) protocol. On the right-hand side, it is possible to specify whether images where obtained with or without injection of Gadolinium, in order to improve the results of subsequent segmentation routines.

It allows the importation of all data into the database: images (native or resulting from processing pipelines such as Destrieux’s parcellation, or cortical thickness performed by Freesurfer) in Nifti^5^ and mgz^6^ formats, and SEEG files (only Micromed™ TRC format for now). There is no anonymization process coded in IntrAnat Electrodes, therefore all data must have been anonymized before being imported. When importing the data, IntrAnat Electrodes incorporates the patient identification code specified in the graphical user interface (GUI) in the patient name data field (Figure 2). By default, for each modality three time points are defined, called pre, post and postOp for pre-implantation, post-implantation with the electrodes within the head of the patient, and post-resection surgery. More time points can be easily added, for example to have a full patient case when there is more than one implantation (pre, first implantation, thermocoagulation, second implantation, resection surgery for example). We chose to allow only the three time points by default to adhere to the standard SEEG procedure.

IntrAnat Electrodes then estimates rigid transformations to coregister all images on the 3D T1 pre-implantation using either ANTs or SPM12 routines. To avoid initialization problems, the user is asked to click inside the brain of each imported image. A coarse realignment is then performed before running the coregistration routines. All transformation matrices are saved in the BrainVisa database and are applied by the BrainVisa referential management system. Images are not resampled at this stage, as image resampling is performed dynamically in Anatomist, BrainVisa’s display software.

**Figure 3:**
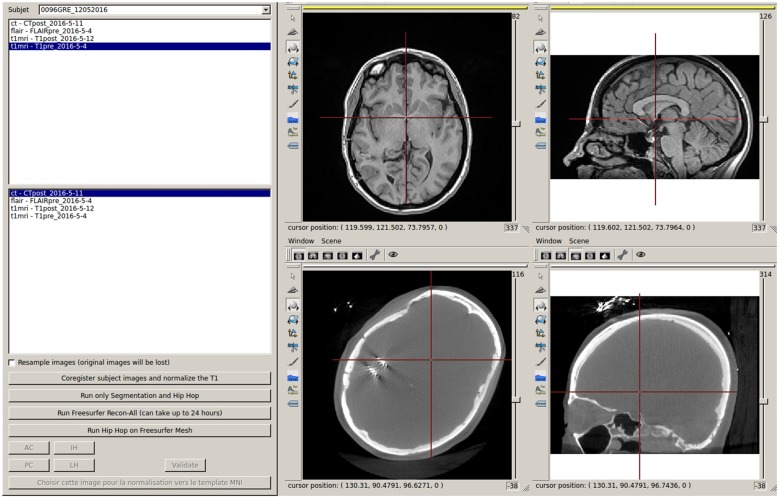
Interface to run the coregistration between imported images and to start MarsAtlas parcellation, Freesurfer recon-all process and MarsAtlas parcellation from Freesurfer gray/white meshes.

From the T1 pre-implantation, IntrAnat Electrodes then estimates the mapping to the MNI referential using SPM12 (Ashburner, 2009) and stores the resulting deformation field in its database. It also runs Morphologist’s gray/white segmentation and sulci recognition (Perrot et al., 2011), and MarsAtlas parcellation (Auzias et al., 2016). If a contrast agent was used to obtain the T1 pre-implantation image, the user has to specify it (Figure 2) in order to allow a specific pre-processing step where IntrAnat Electrodes based on SPM12 will remove all voxels which are not gray or white matter, and then run Morphologist and MarsAtlas on this preprocessed image. If for some reason the SPM12 pre-processing is not satisfactory, IntrAnat Electrodes can use an ANTs commands^7^ (denoising, segmentation of gray/white matter using other MRI modalities than T1 are in development; Avants et al., 2011a,b, 2014). It can also use Freesurfer gray white segmentation which, according to our experience, often fails when a contrast agent is used on the T1, except if we specify as input a T2 or a FLAIR in addition to the T1 (Figure 3).

### Second Interface: LocateElectrodes

**Figure 4:**
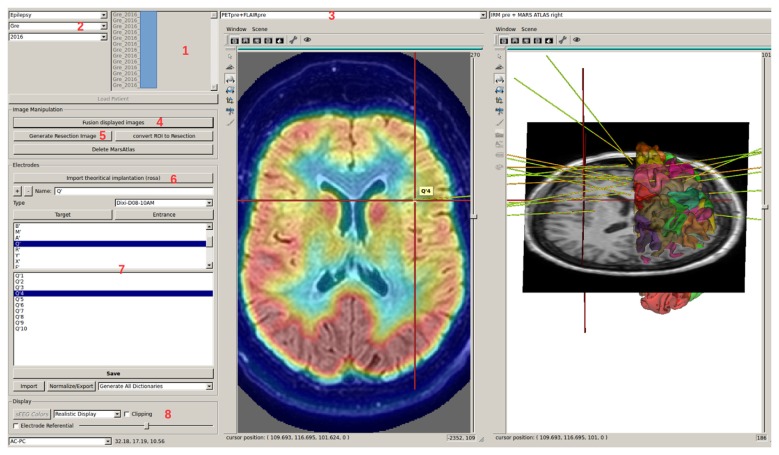
LocateElectrodes interface allows to select a patient in the database (1), using searching criteria (2). The user can navigate in the different imported images using (3), fusion them easily (4), generate the resection mask (5) if the T1 post-resection was imported. Using (6), the user can add, position and name electrodes after choosing a predefined 3D model. Clicking on electrode/contact names (7) updates the 3D Anatomist viewers on the right (here on contact Q’4), and the red 3D cursor moves to the contact. The view can be axial/sagittal/coronal, or in the electrode referential using (8). Here, Positron Emission Tomography (PET) and T1 MRI fusion are displayed on the left, MarsAtlas cortical parcellation and T1 MRI on the right with the implanted electrode models.

**Figure 5:**
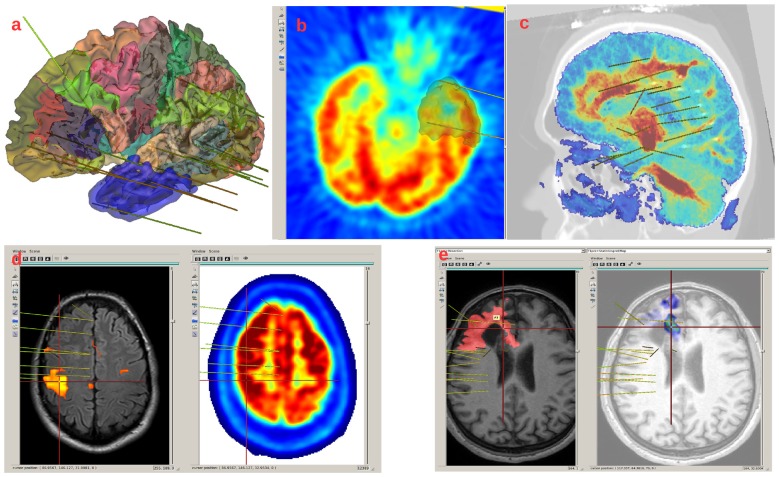
Example of images displayed in LocateElectrodes interface. **(A)** MarsAtlas parcellation on the white matter mesh, with the mesh of the resection in transparent blue. **(B)** PET and resection mesh in gray. **(C)** Fusion of the T1MRI and the Diffusion Tensor Imaging (DTI) reconstruction (the colormap codes for the number of fibers, obtained from FSL software), **(D)** fMRI result on the left, PET on the right, **(E)** T1 and resection mask in red on the left, fusion of the T1 and the Epileptogenicity Index map on the right.

**Figure 6:**
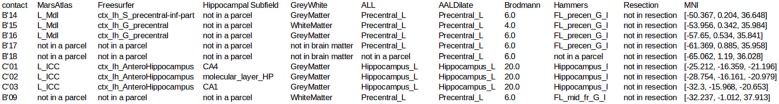
Example of electrode contact labels generated by IntrAnat. Each contact is labeled in all anatomical atlases.

LocateElectrodes provides the patient specific multi-modal data visualization and positioning of electrodes (Figures 4, 5). The software uses 3D rigid models of the electrodes which were created according to manufacturer specifications of electrodes (Dixi Medical, Alcis ADTech, Medtronic, HKHS Healthcare, etc.). The electrodes are manually positioned by users by choosing a model in the available list and by clicking on the post-implantation images on the end of the electrode and any other point along its length. Because pre-implantation and post-implantation images are co-registered, the electrodes can then be visualized in the pre-operative segmented images which are used to label contacts. The software can label each electrode contact automatically according to its position in the anatomical atlases (MarsAtlas, Destrieux, Brodmann, AAL or MaxProbMap), and to its presence in the resection mask (Figure 6). Each electrode contact is assigned to the gray or white matter and to specific anatomical parcels with the following procedure: the most common label voxel in a sphere of 3 mm radius around the contact center is set as the contact label. For cortical atlases, it takes into account only label voxels which are in gray matter. So even if most of the voxel of the sphere are in white matter, it will assign a cortical label if there are some voxels of the label in the sphere. It can also set as label the average value of a statistical map around the contact (e.g., sphere size of 3 mm diameter) such as the epileptogenicity map (David et al., 2011) or the PET image value.

**Figure 7:**
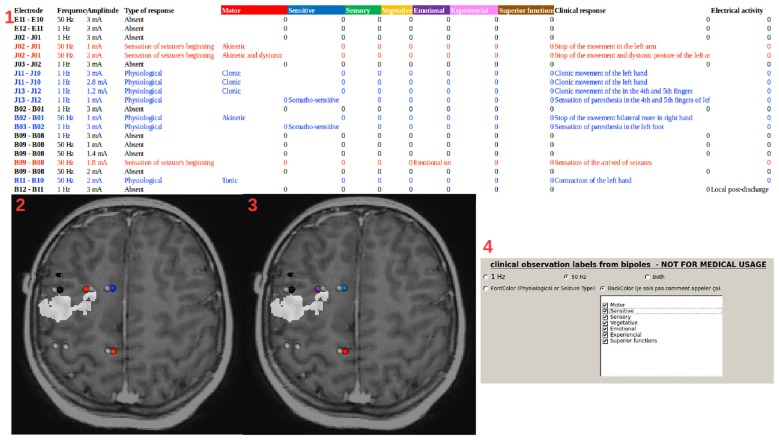
Stimulation labels. (1) Example of a table file (first line and column generated by IntrAnat) filled manually during the stimulation sessions. The file is then read by IntrAnat and integrated into the database. Information can be displayed by LocateElectrodes as in (2) and (3) using the pre-implantation T1 fused with the resection mask (in white) as a background. Using the GUI (4), one can select the information that is displayed (in the case of cortical stimulation here, 1 Hz stimulation, 50 Hz stimulation or both). The font color of the table file is used to set the colormap: non-stimulated contacts in gray, stimulated contacts with no clinical observation in black, stimulated contacts where the patient had a response but without a link to epilepsy in blue, stimulated contacts where the patient had a response similar to the beginning of a seizure in red (2). Alternatively, one can display the classification of the response (using the background color of the line 1 of the table file): motor in red, sensitive in blue, sensory in green, vegetative in yellow, emotional in purple, experiential in pink and superior function in brown (3). The GUI (4) allows to display all or part of these classifications. Contacts not recorded in the SEEG file are not represented.

LocateElectrodes also generates pre-filled table files to easily label contacts manually from the SEEG signals or to add clinical information, such as clinical response after electrical stimulation, cortico-cortical evoked potentials, gamma band activity during cognitive tasks or epileptogenicity during seizures (Figures 6, 7). Once the file is filled, it is converted into Python dictionaries using openpyxl and integrated to the IntrAnat database.

**Figure 8:**
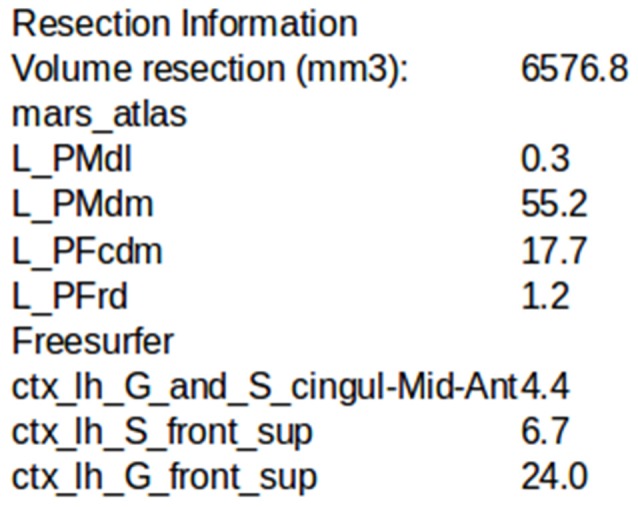
Example of the estimation of the intersection between the resection volume and MarsAtlas/Freesurfer atlas parcels.

A resection mask can be computed on-demand in the software. It is only qualitative and not quantitative as it is sensitive to brain deformation following the surgery. To compute it, the brain mask of the T1 post-resection image is resampled in the T1 pre-implantation referential using the rigid transform computed by ImageImport, as automatic non-rigid registration methods were found not robust enough in this context. It is then subtracted to the pre-implantation brain mask, so all voxels that were identified as gray/white matter in the pre-implantation mask but not in the post-resection mask are kept. A morphological opening is performed to smooth the results. The user then has to click within the main resection volume, and using a connected components function only the component corresponding to where the user clicked will be defined as the resection mask. LocateElectrodes can compute its volume and its overlap with MarsAtlas and/or Destrieux parcels, expressed as the percentage of the parcel’s volume removed by the resection (Figure 8). This procedure does not take into account the post-operative brain deformation, so the percentage is an estimate that can be used in cortical resection cases, but not in case of strong brain deformation as the values may not be robust.

### Third Interface: GroupDisplay

The GroupDisplay interface is used in the context of group studies or to search for patients matching some clinical and/or anatomical criteria. It is composed of two windows.

#### First Window: Patient Fitter Interface

**Figure 9:**
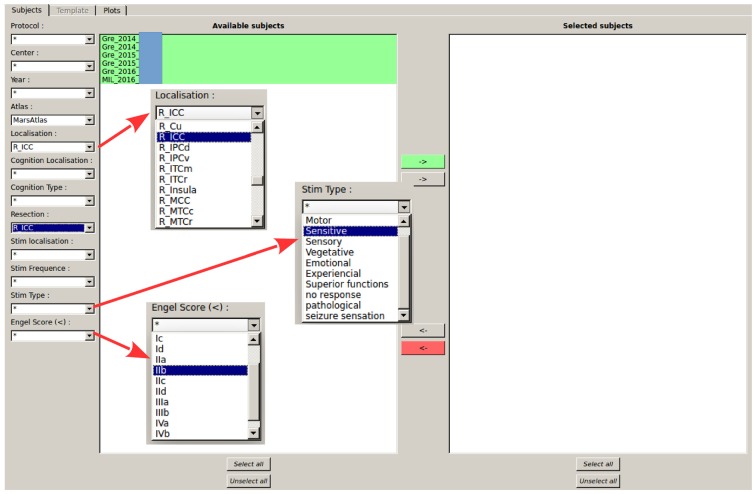
Selection of patients according to labels. All patients from the database validating all criteria selected on the left are shown in the available subject list. Matching criteria on the left-hand side of the GUI are: center and year of acquisition, contact localization according to the parcels (here MarsAtlas parcels, but other atlases can be chosen), cognitive response from SEEG data (still under development), resected parcels, stimulated parcels, stimulation parameters, Engel’s score. In the shown configuration, the software indicates all patients who had an electrode contact in the right isthmus of the cingulate cortex (R_ICC) and for whom the resective surgery included at least partially the right ICC.

This interface uses the contact labels to search for patients in the database according to multiple criteria (contact position, resection localization, response to stimulation, outcome of the resective surgery, cognitive responses, etc.), representing almost all available data (Figure 9). This allows to select a group of patients with similar characteristics, which will be displayed in the second step of the group studies.

#### Second Window: Glass Brain

**Figure 10:**
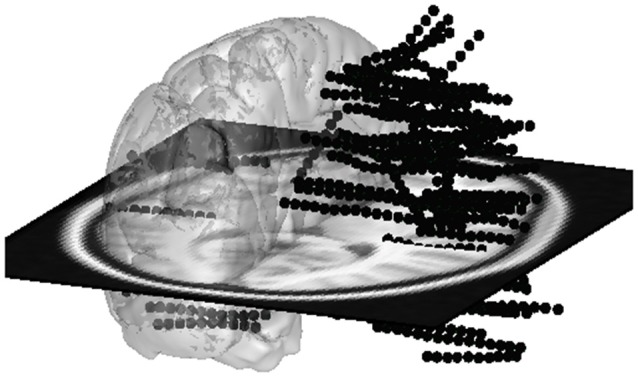
All electrode contacts from four patients superimposed on a glass template brain. If lateralization is not important for the current study, all contacts from the left side can be switched to the right side to combine left and right side.

**Figure 11:**
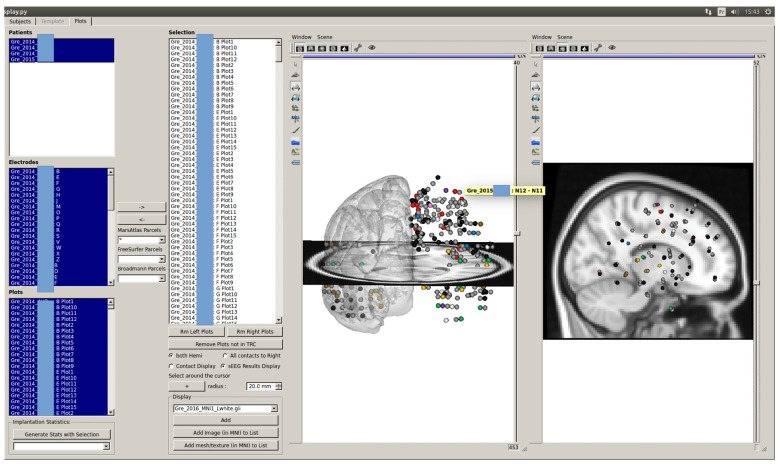
Stimulation results of multiple patients displayed on the glass brain: similarly to the patient visualization in Figure 7, the stimulation results are shown as color-coded spheres. Color code is: gray—bipole not stimulated; black—stimulated but no response; red—motor sensation; blue—sensitive sensation; green—sensory sensation; yellow—vegetative sensation; purple—emotional sensation; pink—experiential sensation; brown—superior function sensation; white—multiple sensations.

**Figure 12:**
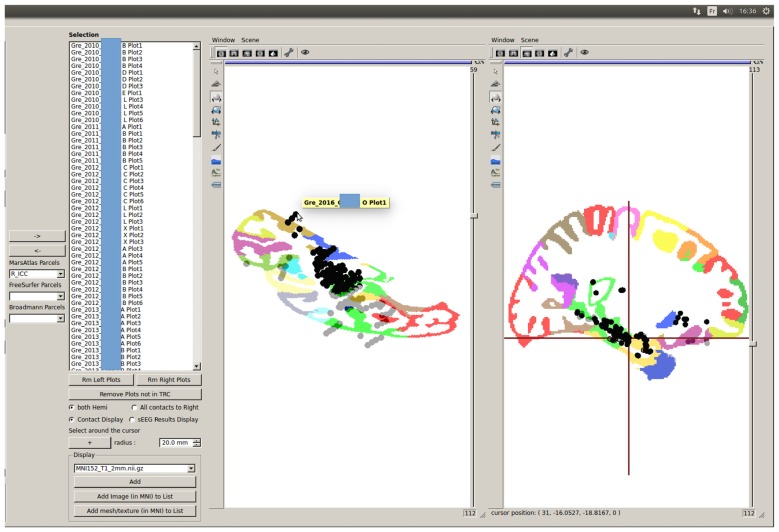
Quick MarsAtlas parcellation quality control based on GroupDisplay of contacts labeled as being from the same parcel. Outliers can be easily identified : here, some contacts from one patient are very far from the ICC region of the template, despite being labeled as ICC contacts. The atlas labeling from this patient must be corrected by tuning the atlas registration parameters for optimized results. An automatic outlier detection tool is available (see Figure 13).

The second window of the group study interface is the glass brain application. It shows all required contacts of the patients selected by the Patient Filter Interface (Figure 10). The contacts can be selected patient by patient, electrode by electrode or one by one (as shown on the left-hand side of Figure 11). Or we can automatically show all (and only contacts) within a MarsAtlas parcel (Figure 12).

Group studies can allow a quick quality control of the MarsAtlas parcellation, which may fail in case of large cortical malformations or bad contrast to noise ratio in T1 images. Figure 12 shows the MarsAtlas parcellation of a template image provided in SPM12 (MNI 152) along with all the contacts supposedly located in the right isthmus of the cingulate cortex (R_ICC). One can see that some contacts are clearly outside the main cluster of contacts in the light green area (R_ICC). Clicking on the mislocated contact shows which patient has to be checked (Figure 12). In that case, the MarsAtlas parcellation should be performed again, validating the process step by step.

**Figure 13:**
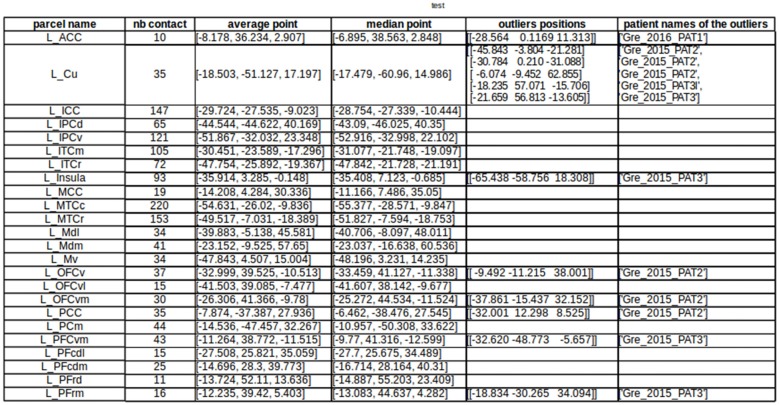
Automatic detection of suspicious parcellation based on the MarsAtlas contact labeling and contact MNI position. This allows quick identification of patients whose atlas labeling failed, by checking the distance between the labeled contacts and the expected location of the region in the MNI template.

Going further in the automatic detection of false parcellations, we have developed in the groupDisplay interface an approach to automatically find patients for whom the parcellation may be incorrect. For each parcel (using MarsAtlas and Freesurfer parcellation), it will estimate the mean and median MNI position of the contacts and the Median Absolute Deviation (MAD = median(X|i − median(X)|), X being the dataset) is calculated. Each patient having a contact further than four times the MAD is specified in a csv file and should be considered as suspicious (Figure 13). A Chauvenet procedure was implemented as well: for each contact position xi, if n*erfc(|xi − mean(x)|/std(x)) < 0.5 then xi defined as suspicious.

## Discussion

The software presented here is, to our knowledge, the first SEEG dedicated software based on a database interface to select patients according to many criteria. It uses well established research neuroimaging toolboxes (BrainVisa, Freesurfer, SPM and ANTs) to recycle optimal and validated image analysis processes. IntrAnat Electrodes requires Matlab for the glass brain group visualization and parcel quality control as, for now, the MNI normalization is performed through SPM12. Moreover, BrainVisa is interfacing with some FMRIB Software Library (FSL^8^) function as well, so that IntrAnat Electrodes is a versatile tool that uses in an optimal way the neuroimaging open platforms in the context of SEEG.

IntrAnat Electrodes is still under development and a certain number of improvements will soon be provided:

1. The forthcoming use of ANTs to perform the normalization should remove the dependency to Matlab. However, group analysis without electrode contacts visualization can be performed using MarsAtlas and Freesurfer parcels as MNI normalization is not required to match parcels between patients. ANTs brain extraction and cortical thickness process should also be callable soon from the IntrAnat interface.
2. The gray/white matter labeling is binary for now: a voxel is in gray matter or in white matter. In order to have a more nuanced labeling, which would better describe if electrode contacts are closer to the gray/white frontier or to the gray/pial frontier, we are implementing a calculation of the distance between the electrode contact and the gray/white matter frontier, normalized by the cortex thickness generated by Freesurfer or ANTs. This procedure is inspired by the gray matter proximity index proposed by Arnulfo et al. (2015a).
3. Nowadays, only rigid electrode models can be used, even if in a few cases the electrodes can be slightly curved due to the implantation process. It will soon be possible to adjust the positions of contacts to the actual curvature of electrodes, in a similar way as in Arnulfo et al. (2015b).
4. The contact labeling according to a series of functional localizers based on induced beta and gamma activity (Vidal et al., 2011) is still under development. The main goal will be here to have a summary in the IntrAnat Electrodes database of the main functions recorded on each electrode contact and cortical parcel.
5. The parcellation quality control will be improved based on geometrical metrics of the parcels. For example, if a patient has a parcel volume much larger than others, and not in a lesioned area, its segmentation will be flagged as suspicious. Pre-implantation lesion detection tools based on multi-modal MRI will be added as well to better define lesioned areas.

In case of MarsAtlas parcelation error, there are few possible solutions. The main errors that we found are due to miss segmentation of the gray/white matter. When the images are of very poor quality (very limited spatial resolution or very poor contrast to noise ratio), there is no solution. On our docker image we have the compiled DenoiseImage from ANTs which can be try to reduce the noise. Otherwise, there is room for playing with some parameters as follows.

By default, Morphologist segmentation and meshes are used. It is possible to use the ones from Freesurfer (it can be done directly from our GUI) and check if the results are better. In general, Freesurfer is particularly useful if the user has T2 or FLAIR because Morphologist does not use these modalities for segmentation purposes.

Using BrainVisa interface it is also possible to modify manually the Gaussian curves used to segment gray and white matter and rerun MarsAtlas segmentation.

If the issue is due to a former brain surgery or lesion, it is possible, using BrainVisa, to define a lesion mask. This part of the brain will be exclude of the Morphologist and MarsAtlas process and can in the parcelation process of the surrounding area.

IntrAnat Electrodes is already installed for research purposes only, in several centers across the world and has been used on around 400 patients by our team for electrode positioning and anatomical labeling for research projects. It is mainly used for SEEG but is also compatible with deep brain stimulation (DBS) implantations as it contains several models of DBS electrodes. Electrode models are also being extended for grids and strips. IntrAnat Electrodes is freely distributed and feedback from users will be essential to optimize its functionalities.

## Author Contributions

PD, MB, DR, YC, PK and OD designed the software. PD, MB, FT, DR and YC developed the software. PD, MB, FT, A-SJ and OD tested the software. A-SJ and PK provided data used in the report. PD, MB and OD wrote the report.

## Conflict of Interest Statement

The authors declare that the research was conducted in the absence of any commercial or financial relationships that could be construed as a potential conflict of interest.

## Footnotes

^1^http://brainvisa.info

^2^http://brainvisa.info/web/morphologist.html

^3^http://jimbodicomviewer.com/

^4^https://sqlite.org/

^5^https://nifti.nimh.nih.gov/nifti-1/

^6^https://surfer.nmr.mgh.harvard.edu/fswiki/FsTutorial/MghFormat

^7^http://stnava.github.io/ANTs/

^8^https://fsl.fmrib.ox.ac.uk/fsl/fslwiki
